# Segmental contribution to whole-body angular momentum during stepping in healthy young and old adults

**DOI:** 10.1038/s41598-021-99519-y

**Published:** 2021-10-07

**Authors:** Jérémie Begue, Nicolas Peyrot, Angélique Lesport, Nicolas A. Turpin, Bruno Watier, Georges Dalleau, Teddy Caderby

**Affiliations:** 1 Laboratoire IRISSE – EA4075, UFR des Sciences de l’Homme et de l’Environnement, Université de la Réunion, 117 rue du Général Ailleret, 97430 Le Tampon, Ile de la Réunion France; 2 Mouvement - Interactions - Performance, MIP, Le Mans Université, EA 4334, 72000 Le Mans, France; 3 LAAS-CNRS, CNRS, UPS, Université de Toulouse, Toulouse, France

**Keywords:** Biomedical engineering, Health care, Risk factors

## Abstract

Recent evidence suggests that during volitional stepping older adults control whole-body angular momentum (H) less effectively than younger adults, which may impose a greater challenge for balance control during this task in the elderly. This study investigated the influence of aging on the segment angular momenta and their contributions to H during stepping. Eighteen old and 15 young healthy adults were instructed to perform a series of stepping at two speed conditions: preferred and as fast as possible. Full-body kinematics were recorded to compute angular momenta of the trunk, arms and legs and their contributions to total absolute H on the entire stepping movement. Results indicated that older adults exhibited larger angular momenta of the trunk and legs in the sagittal plane, which contributed to a higher sagittal plane H range during stepping compared to young adults. Results also revealed that older adults had a greater trunk contribution and lower leg contribution to total absolute H in the sagittal plane compared to young adults, even though there was no difference in the other two planes. These results stress that age-related changes in H control during stepping arise as a result of changes in trunk and leg rotational dynamics.

## Introduction

There is growing evidence that whole-body angular momentum (H) is tightly controlled in order to safely and efficiently perform activities of daily living^1–4^. H is a mechanical quantity that characterizes the rotational behaviour of the whole body. Precisely, it corresponds to the sum of angular momenta produced by the rotation of all body segments about the body’s center of mass (CoM), i.e., the sum of the local angular momentum (the result of the rotation of the segment about its own CoM) and the transfer term (the result of the relative displacement of the segment to the body’s CoM) of all body segments. This implies that control of H requires appropriate regulation and coordination of rotations of all body segments^2,5^. A small H is typically maintained during walking, i.e., near zero, by cancelling segment-to-segment angular momentum. Importantly, poor H control has been found to be associated with poor balance^6^ and an increased risk of falling during locomotion^7^.

Several works suggest that aging may impair the ability to control H. Pijnappels et al.^7^ showed that, compared with young adults, old adults exhibited an insufficient reduction of H after tripping, reducing considerably the balance recovery success and predisposing them to a fall. More recently, Begue et al*.*^8^ investigated age-related changes in H during volitional stepping. The results of this study revealed that, compared to their younger counterparts, older adults exhibited smaller ranges of H during the double support phase i.e., the anticipatory postural adjustments phase. Conversely, older adults had higher ranges of H during the step execution phase, which could inhibit balance control and potentially impose a higher risk of falling during stepping. Nevertheless, this previous study did not examine whether these age-related changes in H were associated to differences in the angular momenta of individual body segments between young and older adults. Such knowledge could provide a better understanding of the underlying mechanisms of frequent falls during voluntary stepping in the elderly^9^.

Previous studies have also shown that aging leads to a distal-to-proximal redistribution in joint kinetics during walking, with older adults generating more mechanical power and work at the hip joint and less at the ankle joint than younger adults^10–13^. These age-related alterations were ascribed to a strategy implemented by older adults to compensate for ankle muscle weakness. Although this was not verified in the studies, these results could suggest a potentially greater trunk angular momentum contribution to gait in older adults, as the hip joint muscles contribute to trunk movement^14^. This is supported by data revealing that old adults exhibit higher angular motion and velocity of trunk during walking compared to young adults, and that these increases are exacerbated at faster speeds^15,16^. Thus, it could be questioned whether the observed higher ranges of H during stepping are associated to a larger trunk angular momentum.

The aim of this study was to investigate age-related changes in the segment angular momenta and their contributions to H during volitional stepping. We hypothesized that older adults would have a higher H range during stepping compared to young adults due to a larger generation of trunk angular momentum, and as a result, they would exhibit a higher relative trunk contribution to total absolute H during stepping compared to their younger counterparts.

## Methods

### Participants

Thirty-three participants volunteered for this study: 15 healthy young and 18 healthy old adults. Characteristics of the individuals are presented in Table 1. All participants were physically active and did not report having any falls in the previous 12 months before the study began. They were free of neurological, musculoskeletal and other disorders that could affect their normal gait or balance. All volunteers were fully informed of the test procedures and gave their written consent prior to beginning the study. The study protocol was in accordance with the Declaration of Helsinki and approved by the local institutional review board (IRISSE, EA 4075).

**Table 1:** Participant characteristics.

	Young *n* = 15 (9F/6M)	Old *n* = 18 (15F/3M)	*P*-value
Age (years)	25.0 ± 3.20 (19–29)	68.4 ± 4.30 (62–77)	< 0.001
Height (m)	1.70 ± 0.10 (1.55–1.86)	1.58 ± 0.08 (1.47–1.72)	< 0.001
Mass (kg)	62.3 ± 9.50 (44.8–76.1)	58.1 ± 11.6 (37.1–77.1)	*NS*

Values are presented as mean ± SD (range).

*F* female, *M* male, *NS* non-significant difference (P > 0.05).

### Experimental procedure and data collec*tion*

Initially, participants stood barefoot on a first force-plate (60 × 40 cm, AMTI, USA) in a natural upright posture as still as possible with their arms alongside their body. After a verbal “go” signal from the experimenter, stepping was initiated from the first force-plate to the second (100 × 80 cm, SENSIX, France). The second force-plate was located immediately in front of the initial force-plate so that participants could naturally step onto it. Both force-plates, embedded in the walkway, measured ground reaction forces (GRFs) and moments. A total of 49 retro-reflective spherical markers (14 mm diameter) were fixed on bony landmarks of all participants^4,17^: bilaterally on the first and fifth metatarsal heads, second toe tip, calcaneous, lateral and medial malleolus, anterior tibial tuberosity, lateral and medial femoral epicondyles, greater trochanter, posterior and anterior superior iliac spines, acromion, medial and lateral humeral epicondyles, ulnar and radial styloids, second and fifth metacarpal heads, and second fingertip; and a single marker on the processus xiphoideus, incisura jugularis, seventh cervical vertebra, tenth thoracic vertebra, sellion, occiput, vertex, and right and left temporal. A motion capture system equipped with 13 cameras (6 Bonita and 7 Vero Cameras; Vicon, UK) and 2 forces-plates were synchronized using analysis software to simultaneously collect kinematic and force-plate data at 200 Hz and 1000 Hz, respectively.

Individuals were asked to initiate step with their dominant leg (leg used for kicking a ball^18^) and to follow through with their non-dominant leg, stopping on the second force place in a comfortable upright posture. After each trial, participants repositioned themselves in the standardized foot position^19^ marked on the first force-plate. Stepping was performed under two randomized speed conditions: at preferred speed and as fast as possible. Data acquisition was triggered when participants were motionless and at least 1 s before the verbal signal. After familiarization trials, each individual performed 10 trials in each speed condition.

### Skeletal model

A whole-body multi-segment 3D model was used offline to reconstruct the stepping movement. This model consisted of 19 segments (pelvis, torso, head, right and left thighs, shanks, feet, toes, arms, forearms, hands and fingers) and 42 degrees of freedom (dofs).

The whole-body geometric model and lower limb, pelvis and upper limb anthropometry are based on the running model of Hammer et al*.*^20^. Mass properties of the hands were estimated from the regression equations of de Leva et al*.*^21^. The anthropometric description of the torso and head (including the neck) segments were estimated from Dumas et al*.*^22^*.* Each lower extremity consisted of seven degrees of freedom: the hip is modelled as a ball-and-socket joint (3 dofs: flexion–extension, adduction-abduction and rotation), the knee with 1 dof is modelled as a revolute joint (flexion–extension), the ankle is modelled as 2 revolute joints (2 dofs: flexion–extension and inversion-eversion) and toes as one revolute joint at the metatarsals (1 dof: flexion–extension). The pelvis joint is modelled as a free flyer joint (6 dofs) to be able to translate and rotate the model in 3D space. The lumbar motion^23^ and neck joint are each modelled as a ball-and-socket joint (3 dofs: flexion–extension, lateral bending and rotation). Each arm consisted of 8 degrees of freedom: the shoulder is modelled as a ball-and-socket joint (3 dofs: flexion–extension, adduction-abduction and rotation), the elbow and forearm rotation are each modelled as revolute joints^24^ (flexion–extension and pronation-supination, respectively), the wrist flexion–extension and radial-ulnar deviation are modelled as revolute joints and fingers are modelled with one revolute joint (flexion–extension).

### Data analysis

Kinematic and force-plate data were low-pass filtered using a zero lag fourth order Butterworth filter with a 6 Hz and 10 Hz cut-off frequency, respectively. The optimal cut-off frequencies were determined from a residual analysis^25^. The inverse kinematics problem was solved with OpenSim software^26^ by minimizing the squared three-dimensional distances between the measured marker locations and the model’s virtual marker locations, subject to the joint constraints and motion ranges of the skeletal model^20,27^. The resulting subject-specific kinematics data i.e., positions, orientations (body-fixed X–Y–Z Euler angles) and velocities of body segments, and the inertial parameters were exported to a custom-made Matlab program (Matlab R2020a, The MathWorks, INC., Natick, MA, USA) for calculation of the angular momentum.

The whole-body angular momentum (H) about the body’s CoM position was calculated in the three dimensions from the following equation:

1 $$\overrightarrow{H}=\sum_{j=1}^{n}\left[\left({\overrightarrow{\mathrm{r}}}_{\mathrm{j}}-{\overrightarrow{\mathrm{r}}}_{\mathrm{CoM}}\right)\times {\mathrm{m}}_{\mathrm{j}}\left({\overrightarrow{\mathrm{v}}}_{\mathrm{j}}-{\overrightarrow{\mathrm{v}}}_{\mathrm{CoM}}\right)+{\mathrm{I}}_{\mathrm{j}}{\overrightarrow{\upomega }}_{\mathrm{j}}\right],$$

where n is the number of segments (n = 19); ${\overrightarrow{\mathrm{r}}}_{\mathrm{j}}$ and ${\overrightarrow{\mathrm{r}}}_{\mathrm{CoM}}$ are the position vectors of the CoM of the j-th body segment and the whole-body CoM (the weighted sum of each body segment’s CoM) in the laboratory frame, respectively; ${\overrightarrow{\mathrm{v}}}_{\mathrm{j}}$ and ${\overrightarrow{\mathrm{v}}}_{\mathrm{CoM}}$ are the velocity vectors of the j-th body segment and the whole-body in the laboratory frame, respectively; ${\mathrm{m}}_{\mathrm{j}}$ and ${\mathrm{I}}_{\mathrm{j}}$ are the mass and inertia tensor of the j-th body segment at the CoM, respectively; and ${\overrightarrow{\upomega }}_{\mathrm{j}}$ is the angular velocity vector of the j-th body segment about its CoM. H was expressed in an orthogonal coordinate system, in which the anteroposterior axis (X axis) was directed forward, the vertical axis (Y) was directed upward and the mediolateral axis (Z) was to the right. In order to decrease between-subject variability due to their anthropometric characteristics, H (dimensionless) was normalized by the product of participant’s height, mass and $\sqrt{g\cdot l}$ ($g=9.81 \mathrm{m} {\mathrm{s}}^{-2}$ and $l$ = the person’s height)^8,28^.

### Temporal event detection

Several temporal events of the stepping movement were determined from kinematic and force-plate data. The onset of the movement (t_0_) was detected when one of the anteroposterior and mediolateral accelerations of the CoM deviated 2.5 standard deviations from its baseline value^8,29^. Foot-off of the dominant leg (FO_D_) was determined as the instant when the vertical position of the fifth metatarsal marker fixed on the dominant leg increased by 5 mm from its mean position during the initial upright posture. Foot-contact of the dominant leg (FC_D_) and foot-off of the non-dominant leg (FO_ND_) were detected when the vertical force signal of the second force-plate exceeded 10 N and the vertical force signal of the first force-plate fell below 10 N, respectively. Foot contact of the non-dominant leg (FC_ND_) was determined from the anteroposterior velocities of the calcaneus and the second toe marker of the non-dominant leg. Precisely, this event corresponded to the instant at which one of these velocities deviated 2 standard deviations from its final baseline value. The end of the stepping movement (T_f_) was defined as the time-point at which the mediolateral CoM velocity remained within 2 standard deviations of the mean calculated during the terminal-stance quiet standing after the end of the stepping^30^.

### Dependent variables

**Figure 1:**
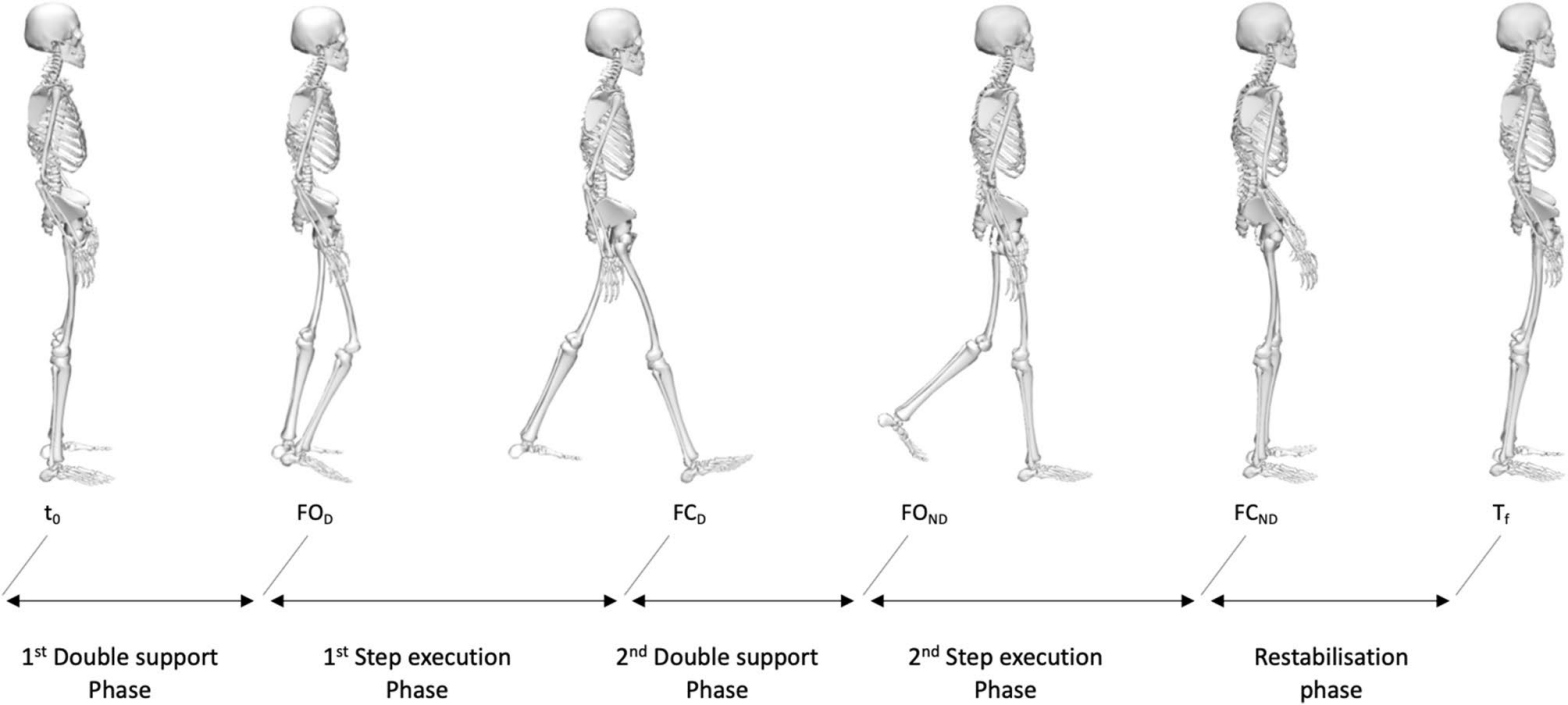
Representation of the step execution movement phases. *t*_*0*_ the onset of the movement, *FO*_*D*_ foot-off of the dominant leg, *FC*_*D*_ foot-contact of the dominant leg, *FO*_*ND*_ foot-off of the non-dominant leg, *FC*_*ND*_ foot-contact of the non-dominant leg, *T*_*f*_ the end of the stepping movement.

In the current study, we investigated the entire stepping movement: two double support phases, two step execution phases and one restabilisation phase (Fig. 1). The first double support phase corresponded to the time between t_0_ and FO_D_. The second double support phase corresponded to the time between the instants of FC_D_ and FO_ND._ The first step execution phase was determined as the time delay between FO_D_ and FC_D_ and corresponded to the swing phase of the dominant leg. The second step execution phase was determined as the time delay between FO_ND_ and FC_ND_ and corresponded to the swing phase of the non-dominant leg. The restabilization phase was determined as the time between FC_ND_ and T_f_. In addition to temporal parameters, forward progression velocity (peak anteroposterior CoM velocity during stepping) and spatial parameters, such as length and width of the first step (the step of the dominant leg) and the second step (the step of the non-dominant leg), were computed. To provide non-dimensional measures, spatiotemporal parameters were normalized by the subject’s body size according to Hof^31^.

Peak-to-peak ranges of H in the three dimensions, determined as the difference between maximum and minimum values of H, were computed over the entire duration of the stepping movement (Total H range) and also in each phase to quantify variation in H.

Angular momentum about the total body CoM was calculated for arms (sum of the instantaneous angular momenta of the upper-arms, forearms, hands and fingers), legs (sum of the instantaneous angular momenta of the thighs, shanks, feet and toes) and trunk (sum of the instantaneous angular momenta of the head, torso and pelvis) during stepping movement. The absolute angular momentum of each of these three segments was averaged over the total duration of the stepping movement about X, Y and Z axes. In addition, we expressed segmental contribution of the arms, legs and trunk as a percentage of the averaged absolute total angular momentum (sum of the average absolute angular momentum of all segments) to determine the relative importance of the angular momentum of each segment (relative contribution of the segments to total absolute H)^32^.

### Statistical analysis

All dependent variables were averaged over ten trials for both speed conditions, per adult. Two-factor (Group $\times$ Speed) mixed-model ANOVAs were performed to evaluate the effects of both the group (between-subjects factor) and speed (within-subjects factor) and their interaction on all dependant variables. For each ANOVA, partial eta-squared value (η_p_^2^) was presented as a measure of effect size. Tukey’s post hoc analysis was conducted when a significant effect was reported. The level of statistical significance was set at *P* < 0.05. The statistical analysis was performed using Statistica software (version 8.0, StatSoft Inc, Tulsa, USA).

## Results

### Ranges of H

**Figure 2:**
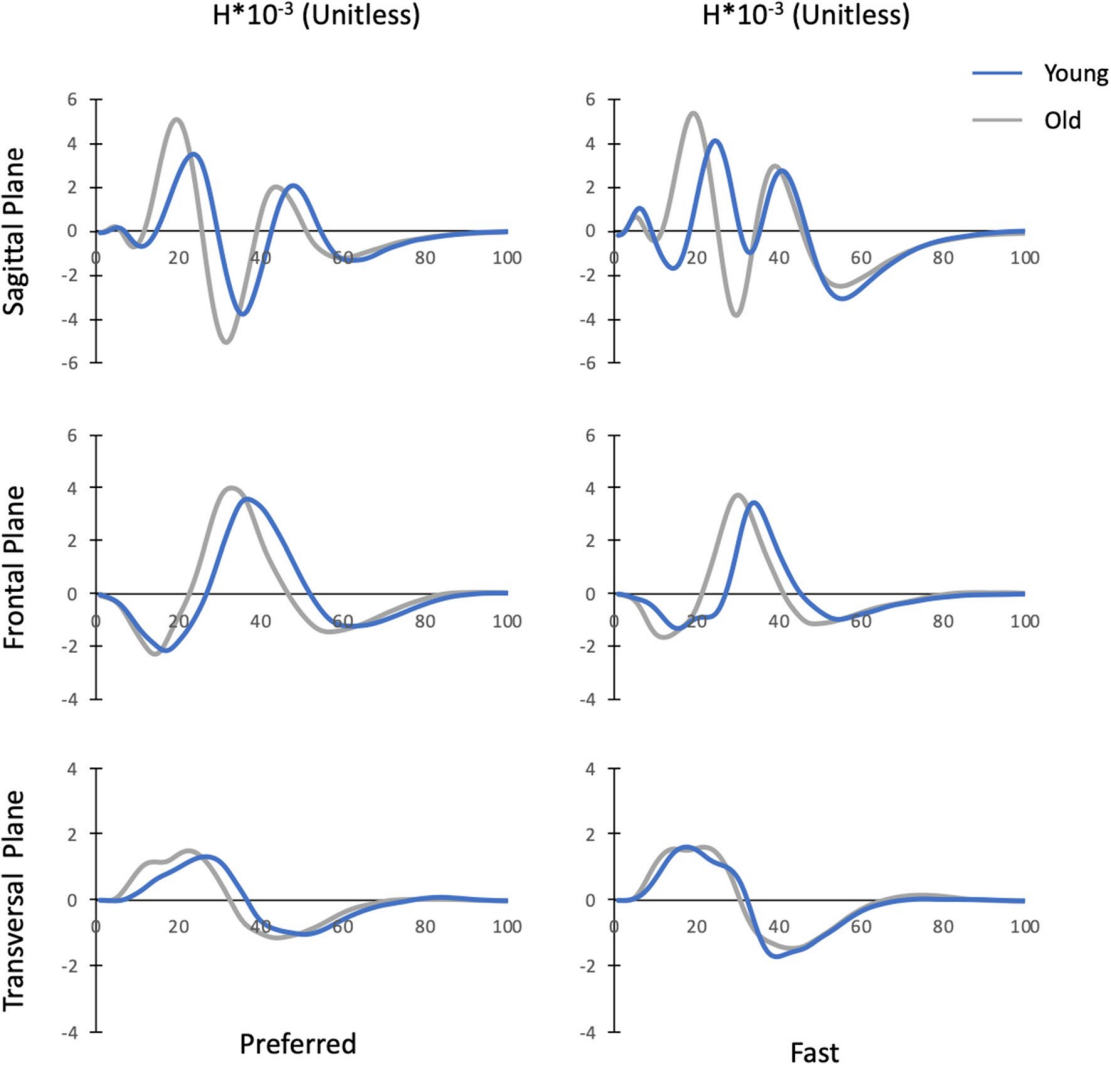
Mean normalized whole-body angular momentum (H) over the entire stepping movement (0–100%) for old and young individuals in both speed conditions. H was normalized by body mass, body height and $\sqrt{g\cdot l}$ ($g$ = 9.81 m s^−2^ and $l$ = body height).

**Figure 3:**
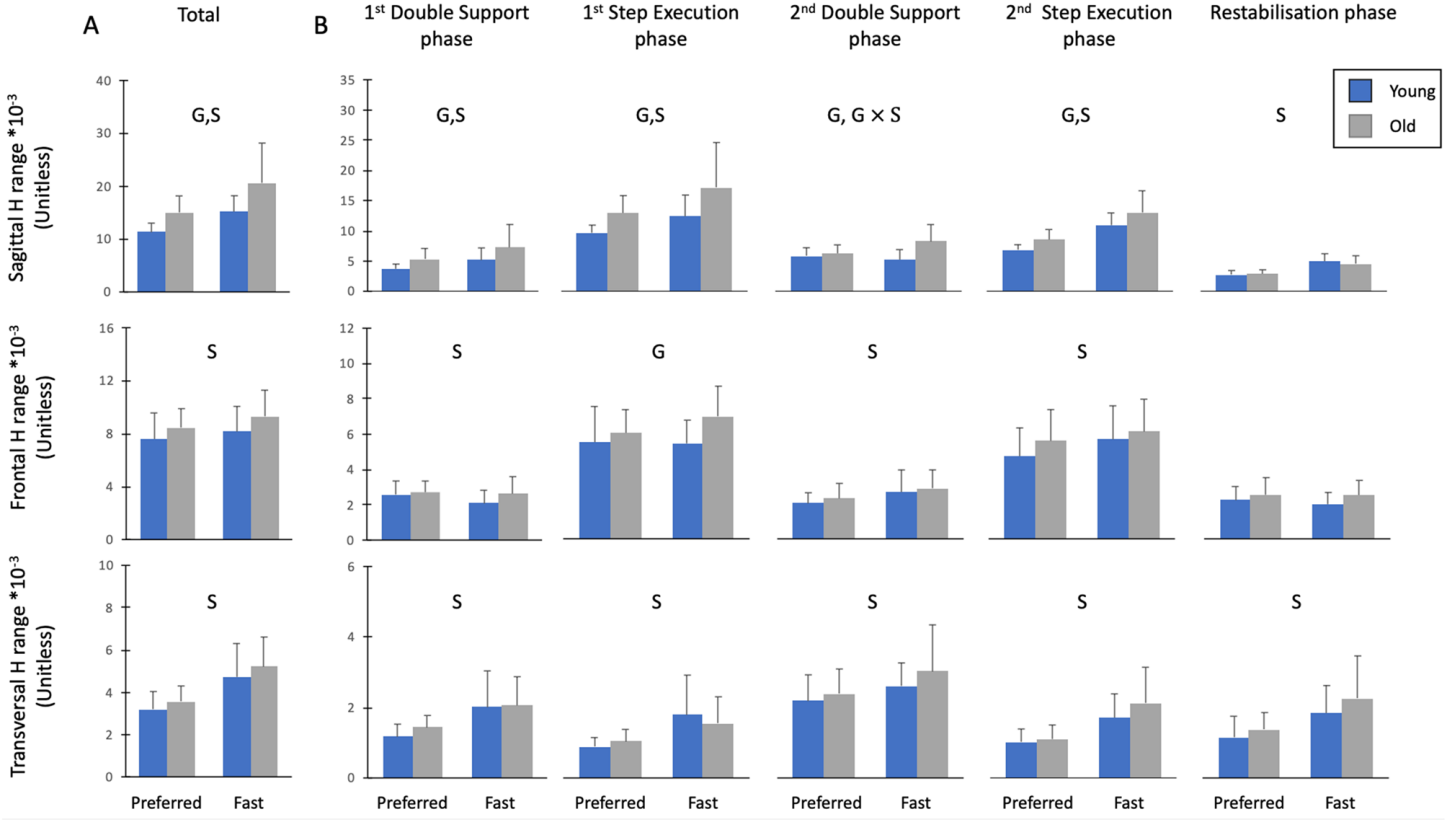
The sagittal, frontal and transversal mean values ± SD of ranges of normalized whole-body angular momentum (H) for old and young individuals. Mean ranges of normalized H were presented for the preferred and fast conditions. H was normalized by body mass, body height and $\sqrt{g\cdot l}$ ($g$ = 9.81 m s^-2^ and $l$ = body height). (**A**) H range computed over the entire duration of the movement (Total H range). (**B**) Ranges of H in frontal, transversal and sagittal planes for all stepping phases. ‘G’ indicated significant differences between old and young individuals. ‘S’ indicated significant differences between the preferred and fast speed conditions. ‘$\mathrm{G}\times \mathrm{S}$’ indicated significant Group $\times$ Speed interaction.

Time evolution of H and ranges of H for young and old adults in both speed conditions are presented in Figs. 2 and 3, respectively.

#### Sagittal plane

A significant Group $\times$ Speed interaction effect was found in the sagittal plane for H range in the second double support phase (*P* < 0.01, η_p_^2^ = 0.26). Compared to young adults, the H range of old adults increased with speed (*P* < 0.01). In addition, H range was significantly higher in older adults for the fast condition (*P* < 0.001), while it did not differ between both age groups for the preferred condition (*P* > 0.05). A group effect was found for the Total H range (*P* < 0.01, η_p_^2^ = 0.26; Fig. 3A) and H ranges in all stepping phases (*P* < 0.05, η_p_^2^ = 0.17–0.23; Fig. 3B), except for the restabilization phase (*P* > 0.05, η_p_^2^ = 0.007). Older adults exhibited higher sagittal H ranges compared to their younger counterparts. Total H range (*P* < 0.001, η_p_^2^ = 0.52) and H ranges in all stepping phases increased with speed (*P* < 0.001, η_p_^2^ = 0.36–0.78), except in young adults for the second double support phase (*P* > 0.05).

#### Frontal plane

Statistical analysis revealed no Group $\times$ Speed interaction effect and no group effect on the Total H range and H ranges in the different stepping phases (*P* > 0.05, η_p_^2^ = 0.001–0.08; Fig. 3), except in the first step execution phase (*P* < 0.05, η_p_^2^ = 0.14). Old adults exhibited a higher range of H compared to their younger counterparts during this phase. With the increase in speed, Total H range (*P* < 0.01, η_p_^2^ = 0.22) and H ranges in both the second double support phase (*P* < 0.01, η_p_^2^ = 0.28) and the second step execution phase (*P* < 0.001, η_p_^2^ = 0.38) increased, while H range in the first double support phase decreased (*P* < 0.05, η_p_^2^ = 0.14).

#### Transversal plane

There was no group or interaction effect on H ranges in the transversal plane (*P* > 0.05; η_p_^2^ = 0.002–0.05). In contrast, the Total H range (*P* < 0.001, η_p_^2^ = 0.67; Fig. 3A) and H ranges in all stepping phases (*P* < 0.05, η_p_^2^ = 0.16–0.54; Fig. 3B) increased with speed.

The net external moment components (Peak GRFs, moment arms and free vertical moment), defined as quantities that contribute to time rate of H (the equivalent of net external moment about CoM)^33^, were also calculated; results are detailed in supplementary material (Tables S1, S2).

### Segment angular momenta and segmental contributions to absolute H

**Figure 4:**
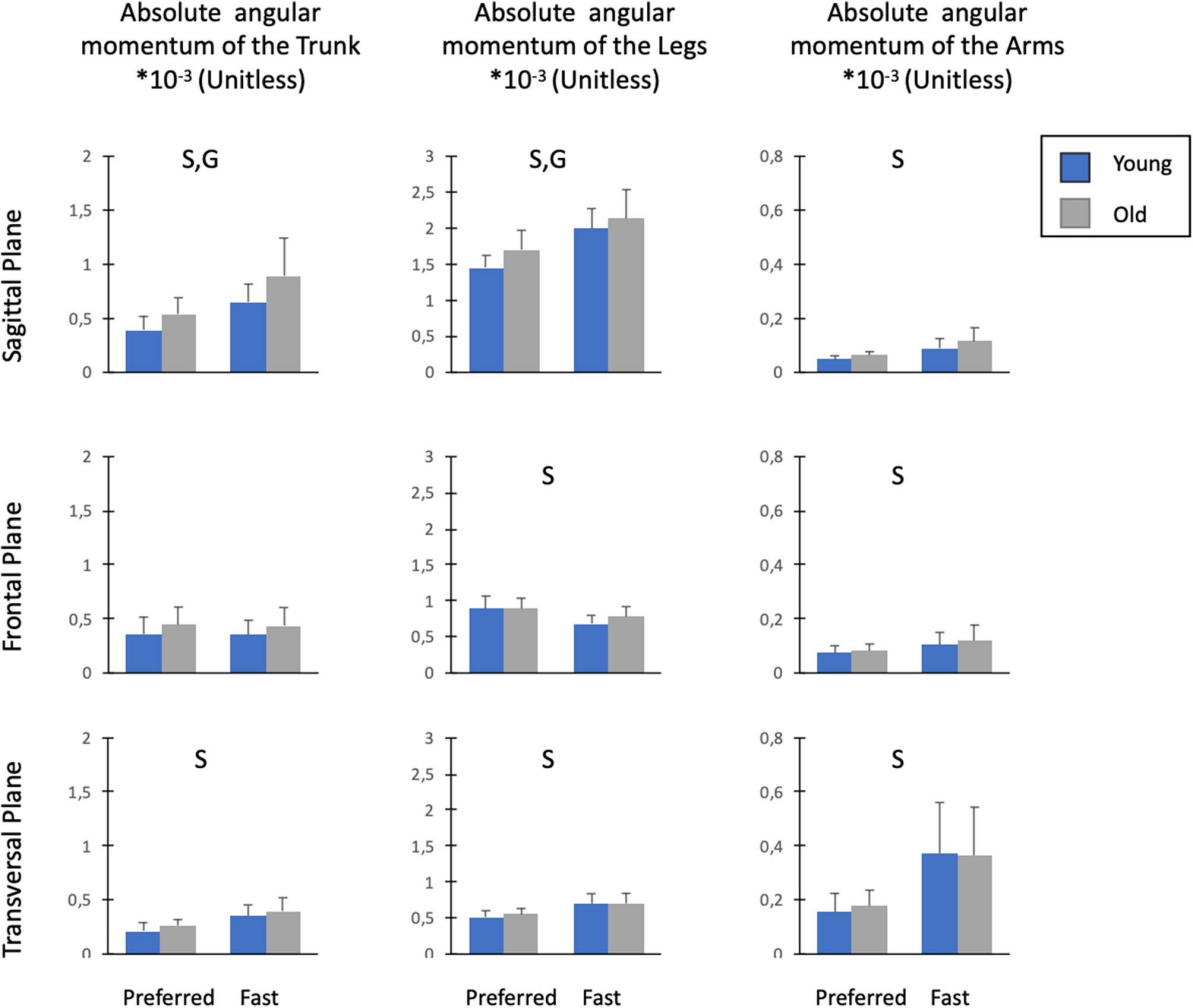
Mean values ± SD of the absolute angular momentum of the trunk, legs and arms in the sagittal, frontal and vertical planes. Absolute angular momentum was averaged over the total duration of the stepping moment and normalized by body mass, body height and $\sqrt{g\cdot l}$ ($g$ = 9.81 m s^−2^ and $l$ = body height). ‘G’ indicated significant differences between old and young individuals. ‘S’ indicated significant differences between the preferred and fast speed conditions. ‘$\mathrm{G}\times \mathrm{S}$’ indicated significant Group $\times$ Speed interaction.

**Figure 5:**
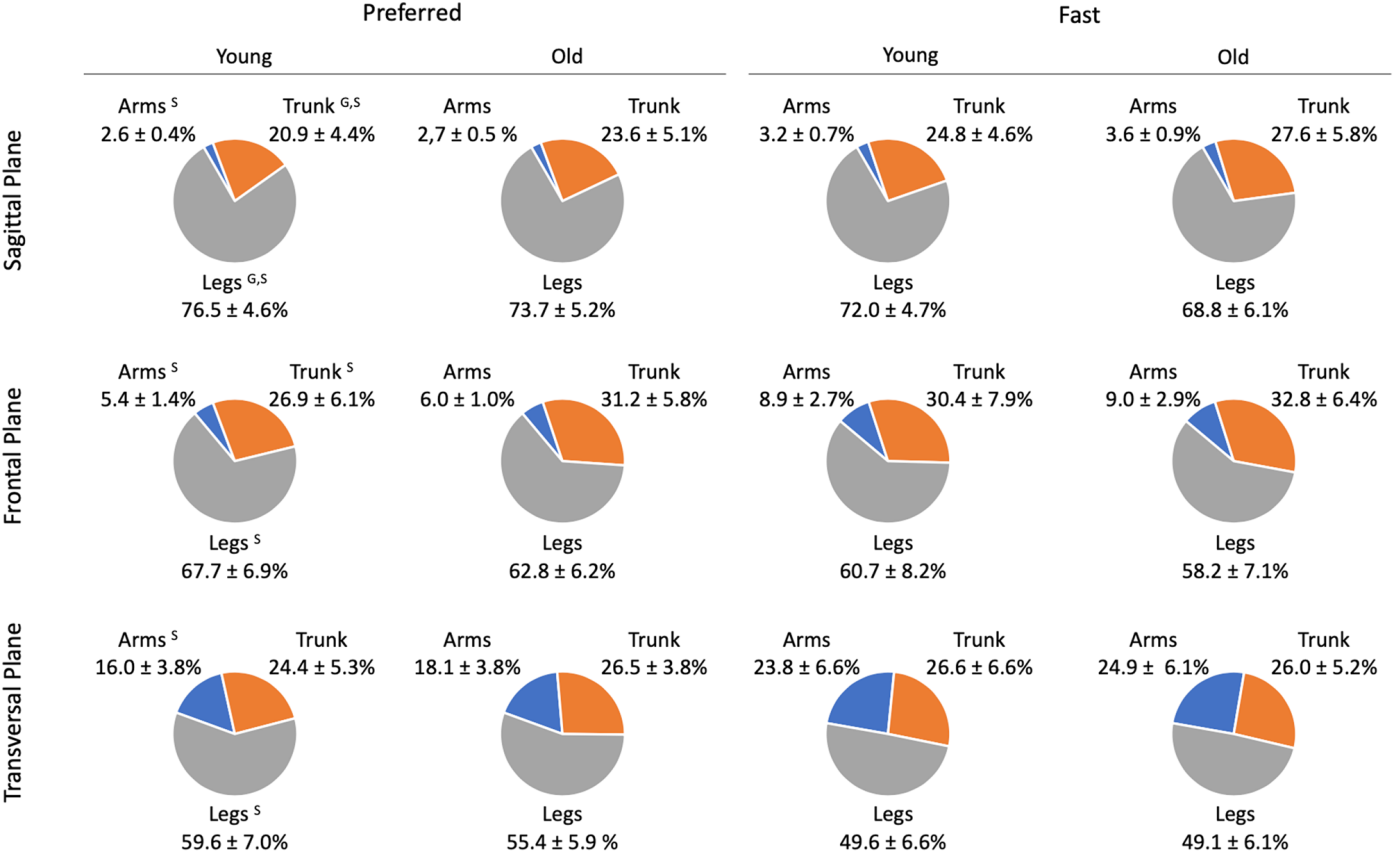
Mean values of the contribution to total absolute H (%) of the trunk, legs and arms in the sagittal, frontal and transversal planes. Relative contributions of the segments were averaged over the total duration of the stepping movement. ‘G’ indicated significant differences between old and young individuals. ‘S’ indicated significant differences between the preferred and fast speed conditions.

Segment angular momenta and segmental contributions to absolute H are detailed in Figs. 4 and 5, respectively.

#### Sagittal plane

No significant Group $\times$ Speed interaction effect was found for the angular momenta of body segments in the sagittal plane (*P* > 0.05, η_p_^2^ = 0.01–0.04). In contrast, there was a significant group effect for the absolute angular momenta of the trunk (*P* < 0.01, η_p_^2^ = 0.22) and legs (*P* < 0.05, η_p_^2^ = 0.13). Compared to younger adults, older participants had higher trunk and leg angular momentum. Furthermore, we found a significant speed effect for angular momenta of the trunk (*P* < 0.001, η_p_^2^ = 0.65), legs (*P* < 0.001, η_p_^2^ = 0.74) and arms (*P* < 0.001, η_p_^2^ = 0.61). The angular momenta of these segments were higher in the fast condition than in the preferred condition.

Regarding the segmental contributions to total absolute H (Fig. 5), the ANOVA revealed no significant Group $\times$ Speed interaction effect in the sagittal plane (*P* > 0.05, η_p_^2^ = 0.003–0.02). In contrast, there was a significant group effect for the trunk (*P* < 0.05, η_p_^2^ = 0.13) and leg (*P* < 0.05, η_p_^2^ = 0.14) contributions. Compared to younger adults, older participants had higher trunk contribution and smaller leg contribution to total absolute H. A speed effect was found for the trunk (*P* < 0.001, η_p_^2^ = 0.46), leg (*P* < 0.001, η_p_^2^ = 0.53) and arm (*P* < 0.001, η_p_^2^ = 0.54) contributions. The trunk and arm contributions increased significantly with speed, while the leg contribution decreased.

#### Frontal plane

Statistical analysis revealed no effect of group or interaction for the angular momenta of all segments in the frontal plane (*P* > 0.05, η_p_^2^ = 0.0003–0.11). Conversely, there was a speed effect for the absolute angular momenta of the arms (*P* < 0.001, η_p_^2^ = 0.43) and legs (*P* < 0.001; η_p_^2^ = 0.64). With the increase in speed, arm angular momentum increased while leg angular momentum decreased.

Regarding the contributions of the segments to total absolute H (Fig. 5), no group or interaction effect was found in the frontal plane (*P* > 0.05, η_p_^2^ = 0.0001–0.08). Nevertheless, a significant speed effect was revealed for the trunk (*P* < 0.01, η_p_^2^ = 0.26), leg (*P* < 0.001, η_p_^2^ = 0.64) and arm (*P* < 0.001, η_p_^2^ = 0.64) contributions. While the contribution of the arms and trunk increased with speed, the contribution of the legs decreased.

#### Transversal plane

No effect of group or interaction was found for the angular momenta of body segments in the transversal plane (*P* > 0.05, η_p_^2^ = 0.001–0.07). In contrast, a speed effect was found for angular momenta of the trunk (*P* < 0.001, η_p_^2^ = 0.74), legs (*P* < 0.001, η_p_^2^ = 0.74) and arms (*P* < 0.001, η_p_^2^ = 0.67). The angular momenta of these segments increased with speed.

Regarding the segmental contributions to total absolute H (Fig. 5), there was no group or interaction effect (*P* > 0.05, η_p_^2^ = 0.0001–0.07). Nevertheless, there was a significant speed effect for leg (*P* < 0.001, η_p_^2^ = 0.72) and arm contributions (*P* < 0.001, η_p_^2^ = 0.74). With the increase in speed, individuals decreased the leg contribution and increased the arm contribution.

The coefficients of angular momentum cancellation for each segment (arms, legs and trunk) in the three planes, reflecting how the angular momentum of components of a given segment cancel each other out, were also computed according to Bennet et al.^5^; results are presented in supplementary material (Table S3).

### Spatiotemporal parameters

Main and interaction effects of normalized spatiotemporal parameters are presented in Table 2. Non-normalized values of spatiotemporal parameters are presented in supplementary material (Table S4). ANOVA results revealed significant speed (*P* < 0.001, η_p_^2^ = 0.86) and Group $\times$ Speed interaction (*P* < 0.01, η_p_^2^ = 0.29) effects for the forward progression velocity, but no group effect for this variable (*P* > 0.05, η_p_^2^ = 0.01). Forward progression velocity increased with stepping speed, but did not differ between young and old adults in both speed conditions. A significant speed effect was also found for all spatial parameters (*P* < 0.05) except for the width of the second step (*P* > 0.05, η_p_^2^ = 0.06). Lengths of the first (η_p_^2^ = 0.60) and second steps (η_p_^2^ = 0.63) and the width of the first step (η_p_^2^ = 0.12) increased with speed. Regarding the temporal parameters, a significant Group $\times$ Speed interaction effect was found for the duration of the second double support phase (*P* < 0.01, η_p_^2^ = 0.22), revealing that this duration decreased in both groups with speed (Table 2). A significant group effect was found for the durations of both the second step execution phase (*P* < 0.01, η_p_^2^ = 0.25) and restabilization phase (*P* < 0.001, η_p_^2^ = 0.41). Compared to younger participants, old participants exhibited longer durations of these two phases. A speed effect was found for all stepping phases (*P* < 0.001, η_p_^2^ = 0.56–0.86), except for the restabilization phase (*P* > 0.05, η_p_^2^ = 0.001). The durations of these phases decreased with speed.

**Table 2:** Normalized spatiotemporal parameters for young and older participants in the preferred and fast speed conditions.

Parameters (dimensionless)	Preferred		Fast		Effect
	Young	Old	Young	Old	
Forward progression velocity	0.17 ± 0.03^a^	0.19 ± 0.03^b^	0.29 ± 0.04^a^	0.26 ± 0.05^b^	S, G $\times \,\mathrm{S}$
Length of the 1st step	0.34 ± 0.05	0.37 ± 0.04	0.43 ± 0.06	0.43 ± 0.09	S
Width of the 1st step	0.10 ± 0.01	0.11 ± 0.02	0.11 ± 0.02	0.11 ± 0.03	S
Length of the 2nd step	0.37 ± 0.06	0.40 ± 0.05	0.47 ± 0.06	0.47 ± 0.10	S
Width of the 2nd step	0.10 ± 0.02	0.10 ± 0.02	0.11 ± 0.02	0.10 ± 0.03	–
1st Double support phase duration	1.49 ± 0.24	1.30 ± 0.21	1.18 ± 0.21	1.10 ± 0.22	S
1st Step execution phase duration	1.04 ± 0.12	1.01 ± 0.13	0.82 ± 0.09	0.85 ± 0.11	S
2nd Double support phase duration	0.70 ± 0.18^a^	0.63 ± 0.12^b^	0.32 ± 0.08^a^	0.39 ± 0.07^b^	S, G $\times \,\mathrm{S}$
2nd Step execution phase duration	1.10 ± 0.12	1.24 ± 0.16	0.86 ± 0.10	0.96 ± 0.12	G, S
Restabilisation phase duration	3.41 ± 0.27	3.88 ± 0.39	3.41 ± 0.35	3.91 ± 0.45	G

Values are presented as mean ± SD.

Velocity was normalized by $\sqrt{g\cdot l}$, distances were normalised by $l$ and durations were normalised by $\sqrt{l/g}$. With $g=$ 9.81 m s^−2^ and $l$ = body height (m). Absence of group, speed and interaction effects (*P* > 0.05) was indicated by ‘_’. Significant group effect is indicated with ‘G’. Significant speed effect is indicated with ‘S’. ‘$G \times S$’ indicated significant Group $\times$ Speed interaction.

Means sharing similar letters differ significantly.

## Discussion

This study aimed to investigate the age-related changes in the segment angular momenta and their contributions to total absolute H during volitional stepping. Consistent with our hypotheses, our main result showed that older adults exhibited a larger variation of trunk angular momentum and a greater trunk contribution to total absolute H in the sagittal plane during stepping, compared to young adults. The larger trunk angular momentum in older adults was associated with a higher total sagittal H range during stepping compared to their younger counterparts. Unexpectedly, our results revealed that the higher H range in the sagittal plane in older adults was also associated to a larger variation of angular momentum of the legs compared to younger adults, while the contribution of the legs to total absolute H during stepping decreased with aging. These results are further discussed in the following paragraphs.

Begue et al.^8^ demonstrated that aging altered the control of H during the initiation phase of stepping, i.e. the phase ranging from the onset of the stepping movement to the foot-contact of the dominant leg. The study also revealed that older adults exhibited smaller H ranges in the sagittal and transversal planes during the (first) double support phase, but higher ranges of H in the sagittal and frontal planes during the (first) step execution phase, compared to young adults. In the current study, we, too, found that older adults had higher ranges of H in the sagittal and frontal planes during the first step execution phase compared to their younger counterparts. In contrast to this previous study, we observed a higher sagittal plane H range during the first double support phase in older participants compared to younger participants and revealed that aging also altered H control in the sagittal plane during the termination phase of stepping (ranging from foot-contact of the dominant leg to the end of stepping), which was not investigated in Begue et al.^8^. While old adults had higher sagittal plane H ranges in both the second double support phase and second step execution phase compared to young adults, there was no difference between both age groups in the restabilization phase. As a consequence of the higher sagittal H ranges in the different phases, old adults exhibited a higher Total H range in the sagittal plane during stepping compared to younger participants (+ 34.7%). These findings support previous assertions that older adults have an impaired ability to control H^7,8,34^.

**Figure 6:**
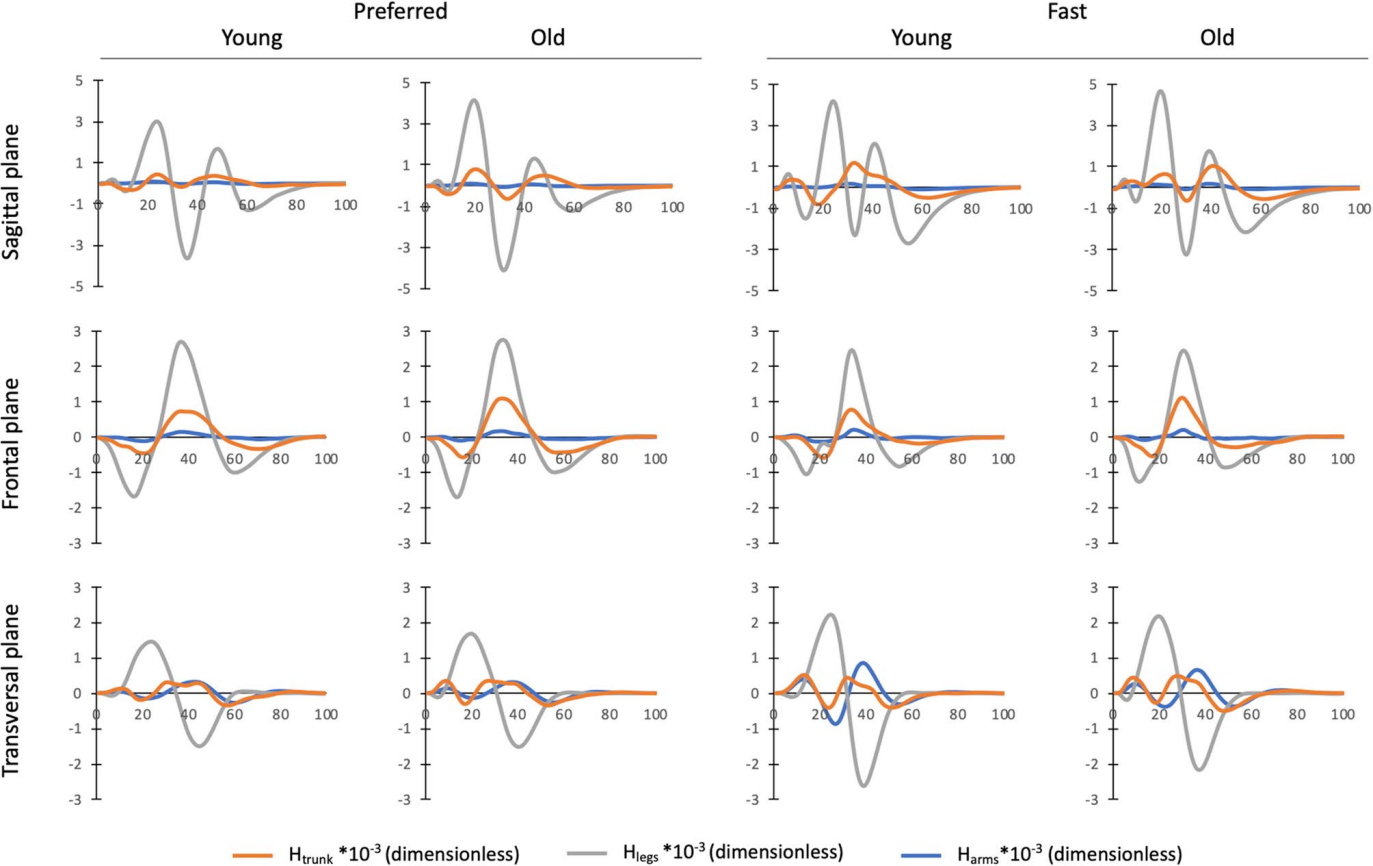
Mean normalized angular momenta of the trunk (H_trunk_), legs (H_legs_) and arms (H_arms)_ over the entire stepping movement (0–100%) for old and young individuals in both speed conditions. Segment angular momenta were normalized by body mass, body height and $\sqrt{g\cdot l}$ ($g$ = 9.81 m s^−2^ and $l$ = body height).

Our results indicate that although age-related differences in H during stepping were mainly accompanied by changes in the segment angular momenta in the sagittal plane, there was no difference between both age groups in the other two planes. Specifically, we found that older adults had larger angular momenta of the trunk (+ 36.9%) and legs (+ 11.1%) in the sagittal plane compared to their younger counterparts. It is worth noting that the angular momenta patterns of the trunk, legs and arms in the sagittal plane were roughly in phase (Fig. 6). As the angular momentum of the arms did not significantly differ between both age groups, the larger trunk and leg angular momenta were both responsible for the higher Total H range in the sagittal plane in older adults.

Our finding that trunk angular momentum is greater in sagittal plane in older adults was expected and is consistent with the literature on walking. It has been previously reported that older adults have a higher trunk angular motion and velocity during walking than younger adults^15,16^, supporting the idea of there being a greater magnitude of trunk angular momentum in the elderly. In contrast, the increase in leg angular momentum with aging may be less obvious. Interestingly, previous studies have shown that older adults exhibit changes in lower extremity joint kinematics and kinetics, such as greater range of motion and power at the hip, and decreased ranges of motion and power at the knee and ankle, compared to young adults^10,35,36^. These age-related alterations, which are mainly attributed to ankle plantar flexors weakness and hip flexion contracture^37,38^, could probably contribute to differences in leg angular momentum between young and older adults. Nevertheless, our results revealed that the changes in leg angular momentum between young and older adults were not attributed to the generation of greater individual angular momenta of the lower-extremity bodies (thighs, shanks and feet) in older adults, as the sum of absolute angular momenta of these individual bodies did not differ between both age groups (Table S3). Instead, our results suggest that the larger angular momentum of the legs in older adults was primarily ascribed to lower cancellations between lower-extremity segments compared to young adults (both speed conditions combined: 58.0% vs. 54.8% in young and older adults, respectively; Table S3), which could be the result of age-related alterations in kinematics and kinetics joints described above.

In addition to the age-related changes in the segment angular momenta, we noted that the segmental contributions to absolute H differed between young and older participants in the sagittal plane. Precisely, older individuals exhibited a higher trunk contribution and a smaller leg contribution to absolute H (both speed conditions combined: 25.6% and 71.3% in older adults vs. 22.9% and 74.3% in younger participants for the trunk and legs, respectively) during stepping. It is noteworthy that compared to young adults, leg contribution to absolute H was lower in older adults, despite their larger angular momentum of legs. This may be explained by the fact that the increase in trunk angular momentum with aging is larger than the increase in the angular momentum of legs (both speed conditions combined: + 36.9% for trunk vs. + 11.1% for legs), relative to H. Taken together, these results emphasize that with aging, a leg-to-trunk shift in segmental contributions to absolute H occurs during stepping. This is consistent with findings from previous studies focusing on the effect of age on joints kinetics during walking^10–13^ which reported a distal to proximal redistribution in joint kinetics with age, with older adults generating more power and work at the hip and less at the knee and ankle compared to younger adults. According to these authors, the age-related changes in joint power and work occurred mainly to compensate for the weakness of plantarflexors to produce propulsive forces. Specifically, older adults use hip musculature (flexors and/or extensors) to assist in advancing the leg into swing phase and in propelling the body forward as a means of compensating for reduced ankle plantarflexor power^35^. As underlined by McGibbon^35^, this strategy consisting of increasing the contraction of hip muscles into leg swing phase may alter pelvic mechanics and trunk behaviour. For instance, an increase in hip extensor concentric contraction of the stance leg during the terminal stance may pull the pelvis and trunk backward and assist leg advancement into swing phase. This strategy of the hip to compensate for ankle plantar flexor weakness could potentially explain the age-related changes in the trunk and leg angular momenta and their contributions to absolute H observed in the current study.

Interestingly, our results revealed that the total propulsive forces, i.e., the anteroposterior forces generated during the first double support phase and the first step execution phase, were not altered by aging (Table S2). This supports the hypothesis that age-related alterations in the segment angular momenta and the segmental contributions to absolute H could probably be the result of a strategy by older adults to maintain a magnitude of propulsive forces similar to young adults during the initiation of stepping. In contrast, we observed that older adults exhibited smaller braking forces than younger adults during the termination of stepping, i.e., second double support phase and second step execution phase, particularly in the fast speed condition. This was accompanied by longer durations of these latter phases and the restabilization phase in older adults compared to their younger counterparts. These results are consistent with those of Tirosh and Sparrow^39^, who reported lower braking force generation and longer stopping time in older adults, compared to younger adults, during gait termination. These authors interpreted their results to mean that older adults have a greater difficulty to arrest the motion of the CoM, which could be due to deficiencies in recruiting ankle plantarflexors and hip abductors. In line with these findings, Pijnappels et al.^7^ showed that, compared to young adults, older people were unable to sufficiently reduce H during the push-off phase after tripping, which reduced the recovery success and increased the frequency of falling. According to these authors, this was due to a lower rate of change of moment generation in all support leg joints and a lower peak ankle moment in older adults. Our results suggest that older adults may have a greater difficulty to control H during termination of stepping compared to stepping initiation, and that this is exacerbated when stepping is performed at a faster speed.

We noted that Total H range in all three planes increased with speed. As the increase in speed resulted in a decrease in phase durations, i.e., a reduction in the time application of the net external moment, the increase in Total H ranges was ascribed to higher net external moments. This is supported by changes in the components of the net external moment, i.e., GRFs and moment-arms, in the different phases (Tables S1, S2), which caused higher H ranges in most stepping phases (Fig. 3B). When analyzing the segment angular momenta, we noted that those of the trunk, legs and arms in the sagittal and transversal planes increased with speed, contributing to the increase in Total H ranges in these planes. While the angular momentum of the legs decreased with the increase in speed, the angular momentum of the arms increased, contributing greatly to the larger variation of H in the frontal plane. Additionally, increases in speed resulted in changes in the segmental contributions to absolute H. The contribution of the trunk and arms increased with speed, while the contribution of the legs decreased in the sagittal and frontal planes. In the transversal plane, similar speed-related changes were observed for the arms and legs. Nevertheless, the contribution of the trunk did not change between both speed conditions in this plane. Our results are partially supported by Bruijn et al.^28^, who investigated the effect of gait speed on the segmental contributions to the total absolute body angular momentum in the transversal plane during walking. These authors showed that an increase in walking speed led to an increase in segment angular momenta and changes in the segmental contributions to total absolute H in healthy young adults. Contrary to our results, they did not observe a significant speed effect on the absolute total body angular momentum. However, the p-value was close to the significant (P = 0.01) and probably related to the small number of participants enrolled in their study (n = 9). It should be noted that other studies that assessed the speed-related changes in H revealed a negative correlation between H and progression velocity during walking^5,40^ and running^41^. This discrepancy could be ascribed to the method used for normalizing H values. Unlike the previous studies, we normalized H independent of speed which could be more relevant for considering speed effect on H^42^.

The current study could have practical implications for preventing falls in the elderly. Our results indicate that the larger angular momenta of the trunk and legs in older adults were associated with a higher Total H range in the sagittal plane during stepping compared to young individuals, which could impose a greater challenge for balance control^6,43–45^ and, consequently, a potentially higher risk of falling during stepping in this population. As discussed above, these age-related alterations in the segment angular momenta and their contribution to total absolute H during stepping could be the result of a strategy adopted by older adults in order to compensate ankle plantarflexors weakness. Taken together, these data reinforce the idea that ankle muscle strengthening could be helpful for older adults to improve balance and reduce the risk of falling during locomotion. Previous studies have already highlighted that an increase in plantar flexor strength is strongly correlated with improved balance^46^ and that ankle power training improved functional mobility^47^ in old adults. Nevertheless, it should be noted that the changes in the trunk angular momentum observed in older adults could also be related to an impaired trunk control. Several studies suggest that older adults have greater difficulty controlling momentum and displacement of the trunk during walking^15,16^, which could be mainly due to a decline in muscle and sensorial systems associated with aging^48^. In particular, according to McGibbon^35^, contraction of hip muscles to assist in advancing leg into swing phase may amplify pelvic rotation, thereby requiring trunk muscles to compensate for altered pelvic mechanics. Age-related decline in trunk muscle strength could limit such compensation and therefore contribute to higher sagittal H range in older adults. Thus, it could be worthwhile to also investigate whether interventions aimed to improve trunk control could decrease trunk angular momentum and in turn reduce the H range during stepping in older adults. As an example, with a high adherence rate, core strength training has been shown to be an efficient way to address the trunk control impairment and enhance balance performance in elderly people^49^.

It should be noted that the older participants included in the present study were relatively young (on average 68.4 ± 4.30 years old), healthy and physically active (a minimum of 2 h of recreational physical activity per week). These characteristics of our participants may probably explain the lack of difference in the frontal plane between the two age groups regarding the total H range and the segmental contributions. These results contrast with those obtained in previous studies that revealed poorer mediolateral balance control in older adults than in their younger counterparts^50–52^. Thus, future studies should explore how aging influences the segmental angular momenta and their contribution to H in much older and sedentary participants, i.e., in those present a higher risk of falling. In addition, because we computed the segmental contribution as a percentage of the average absolute total angular momentum, we did not consider information about the direction of the angular momentum of each segment. Previous studies have used principal component analysis (PCA) on the segmental angular momenta^2,5^ or calculated the positive or negative contribution of the segments^53,54^ to obtain this information. Such approaches could be useful to determine how aging influences the segment interaction strategy and provide additional information about age-related changes in the segmental coordination underlying the control of H.

In conclusion, the current study revealed that aging caused an alteration in the segment angular momenta and their contributions to total absolute H during stepping. Older adults exhibited higher trunk contribution and smaller leg contribution to total absolute H in the sagittal plane compared to young adults. In particular, in the sagittal plane, older adults had larger angular momenta of the trunk and legs than younger adults, which led to a greater Total H range. These results stress that the age-related alterations in H control during stepping arise as a result of changes in trunk and leg rotational dynamics. Given that an impaired ability to control H may jeopardize balance control and thus increase the odds of falling, future studies should investigate whether interventions aiming to redistribute segmental contributions e.g., resistance training programs, may reduce H ranges during stepping in old adults.

## Supplementary Information

Supplementary Information.
