# Supplementary material for: Segmental contribution to whole-body angular momentum during stepping in healthy young and old adults
Source: Sci Rep. 2021 Oct 7;11:19969. doi: 10.1038/s41598-021-99519-y (PMC8497562; doi:10.1038/s41598-021-99519-y)
Supplement: Supplementary file 1 — Supplementary Information. [file 41598_2021_99519_MOESM1_ESM.docx]

Supplementary material

Table S1. Normalized peak GRFs and free vertical moment for young and older participants in the preferred and fast speed conditions.

|  | Preferred | | Fast | | Effect |
| --- | --- | --- | --- | --- | --- |
|  | Young | Old | Young | Old |  |
| ***1^st^ Double support phase*** | |  |  |  |  |
| Peak AP GRF | 0.089 ± 0.022^a^ | 0.109 ± 0.029^b^ | 0.194 ± 0.032^a^ | 0.179 ± 0.036^b^ | S, G $\times S$ |
| Peak ML GRF | -0.059 ± 0.009 | -0.070 ± 0.016 | -0.063 ± 0.011 | -0.076 ± 0.021 | G |
| Peak Vertical GRF | 1,079 ± 0.024 | 1,082 ± 0,032 | 1.060 ± 0.040^a^ | 1.098 ± 0.056^a^ | G $\times S$ |
| Peak T_v_ x 10^-3^ | 3.355 ± 0.915 | 4.459 ± 2.033 | 4.439 ± 0.934 | 5.678 ± 2.057 | G, S |
| ***1^st^ Step execution phase*** | |  |  |  |  |
| Peak AP GRF | 0.144 ± 0.048^a^ | 0.163 ± 0.039^b^ | 0.286 ± 0.072^a^ | 0.237 ± 0.057^b^ | S, G $\times S$ |
| Peak ML GRF | 0.092 ± 0.009^a^ | 0.102 ± 0.015^b^ | 0.131 ± 0.024^a^ | 0.119 ± 0.017^b^ | S, G $\times S$ |
| Peak Vertical GRF | 1.079 ± 0.049^a^ | 1.081 ± 0.055^b^ | 1.248 ± 0.093^a,c^ | 1.144± 0.066^b,c^ | G,S, G $\times S$ |
| Peak T_v_ x 10^-3^ | -4.502 ± 1.912 | -6.388 ± 3.685 | -5.723 ± 2.699 | -7.081 ± 3.958 | S |
| ***2^nd^ Double support phase*** | |  |  |  |  |
| Peak AP GRF | -0.140 ± 0.039^a^ | -0.151 ± 0.029^b^ | -0.305 ± 0.080^a,c^ | -0.240±0.078^b,c^ | S, G $\times S$ |
| Peak ML GRF | -0.078 ± 0.019^a^ | -0.074 ± 0.013 | -0.109 ± 0.036^a^ | -0.087 ± 0.025 | S, G $\times S$ |
| Peak Vertical GRF | 1.226 ± 0.064 | 1.273 ± 0.055 | 1.259 ± 0.130 | 1.311 ± 0.101 | - |
| Peak T_v_ x 10^-3^ | 0.078 ± 2.213 | 0.519 ± 1.410 | 3.724 ± 2.659 | 2.586 ± 2.056 | S |
| ***2^nd^ Step execution phase*** | |  |  |  |  |
| Peak AP GRF | -0.052 ± 0.019^a^ | -0.047 ± 0.020^b^ | -0.135 ± 0.025^a,c^ | -0.095±0.018^b,c^ | G, S, G $\times S$ |
| Peak ML GRF | -0.080 ± 0.018^a^ | -0.079 ± 0.012^b^ | -0.130 ± 0.036^a,c^ | -0.102± 0.022^b,c^ | G, S, G $\times S$ |
| Peak Vertical GRF | 1.033 ± 0.022 | 1.056 ± 0.020 | 1.141 ± 0.096 | 1.130 ± 0.076 | S |
| Peak T_v_ x 10^-3^ | 3.717 ± 2.428^a^ | 3.469 ± 1.335^b^ | 8.406 ± 1.847^a,c^ | 6.295± 1.606^b,c^ | G, S, G $\times S$ |
| ***Restabilisation phase*** | |  |  |  |  |
| Peak AP GRF | 0.005 ± 0.004 | 0.005 ± 0.003 | 0.002 ± 0.003 | 0.004 ± 0.002 | - |
| Peak ML GRF | 0.033 ± 0.012 | 0.027 ± 0.007 | 0.037 ± 0.014 | 0.029 ± 0.010 | G |
| Peak Vertical GRF | 1.055 ± 0.025 | 1.060 ± 0.020 | 1.091 ± 0.061 | 1.092 ± 0.044 | S |
| Peak T_v_ x 10^-3^ | -1.680 ± 1.112^a^ | -1.152 ± 0.771 | -0.889 ± 0.764^a^ | -1.110 ± 0.774 | S, G $\times S$ |

Values are presented as mean ± SD.

Peak ground reaction forces (GRFs) were normalized by body weight (BW). Free vertical moment (T_v_) i.e., the frictional torque about the vertical axis originating at the CoP and resulting shear forces between foot and the ground, was normalized by the product of body height ($l$) and body weight. AP: anteroposterior, ML: mediolateral. Absence of group, speed and interaction effects (*P*>0.05) was indicated by ‘_’. Significant group effect is indicated with ‘G’. Significant speed effect is indicated with ‘S’. ‘$G \times S$’ indicated significant Group $\times$ Speed interaction. Means sharing similar letters differ significantly.

Table S2. Normalized peak moment-arms for young and older participants in the preferred and fast speed conditions.

|  | Preferred | | Fast | | Effect |
| --- | --- | --- | --- | --- | --- |
|  | Young | Old | Young | Old |  |
| ***1st Double support phase*** | |  |  |  |  |
| Peak AP moment-arm | 0.039 ± 0.010^a^ | 0.046 ± 0.012^b^ | 0.078 ± 0.014^a^ | 0.071 ± 0.019^b^ | S, G $\times S$ |
| Peak ML moment-arm | 0.036 ± 0.007 | 0.043 ± 0.009 | 0.037 ± 0.008 | 0.049 ± 0.013 | G |
| Peak Vertical moment-arm | 0.538 ± 0.005^a^ | 0.538 ± 0.007^b^ | 0.533 ± 0.006^a^ | 0.536 ± 0.007^b^ | S, G $\times S$ |
| ***1st Step execution phase*** | |  |  |  |  |
| Peak AP moment-arm | 0.089 ± 0.026 | 0.103 ± 0.018 | 0.149 ± 0.030 | 0.145 ± 0.041 | S |
| Peak ML moment-arm | 0.056 ± 0.005^a^ | 0.062 ± 0.008^b^ | 0.071 ± 0.007^a^ | 0.070 ± 0.011^b^ | S, G $\times S$ |
| Peak Vertical moment-arm | 0.525 ± 0.007^a^ | 0.522 ± 0.009^b^ | 0.512 ± 0.011^a^ | 0.516 ± 0.013^b^ | S, G $\times S$ |
| ***2^nd^ Double support phase*** | |  |  |  |  |
| Peak AP moment-arm | 0.092 ± 0.021^a^ | 0.104 ± 0.017^b^ | 0.163 ± 0.032^a^ | 0.144 ± 0.028^b^ | S, G $\times S$ |
| Peak ML moment-arm | 0.046 ± 0.007^a^ | 0.047 ± 0.008^b^ | 0.061 ± 0.010^a^ | 0.053 ± 0.011^b^ | S, G $\times S$ |
| Peak Vertical moment-arm | 0.536 ± 0.008 | 0.536 ± 0.009 | 0.520 ± 0.012 | 0.524 ± 0.014 | S |
| ***2^nd^ Step execution phase*** | |  |  |  |  |
| Peak AP moment-arm | 0.024 ± 0.008^a^ | 0.021 ± 0.010^b^ | 0.060 ± 0.010^a,c^ | 0.040± 0.008^b,c^ | G, S, G $\times S$ |
| Peak ML moment-arm | 0.023 ± 0.007 | 0.016 ± 0.006 | 0.022 ± 0.009 | 0.016 ± 0.010 | G |
| Peak Vertical moment-arm | 0.544 ± 0.004 | 0.546 ± 0.007 | 0.543 ± 0.005 | 0.544 ± 0.007 | S |
| ***Restabilisation phase*** | |  |  |  |  |
| Peak AP moment-arm | 0.001 ± 0.001 | 0.001 ± 0.001 | 0.001 ± 0.001 | 0.002 ± 0.002 | G |
| Peak ML moment-arm | 0.016 ± 0.006 | 0.014 ± 0.003 | 0.017 ± 0.006 | 0.014 ± 0.006 | - |
| Peak Vertical moment-arm | 0.539 ± 0.005^a^ | 0.540 ± 0.006 | 0.538 ± 0.005^a^ | 0.540 ± 0.007 | G $\times S$ |

Values are presented as mean ± SD.

Moment-arms (the distance between CoM and CoP) were normalized by body height ($l$). AP: anteroposterior, ML: mediolateral. Absence of group, speed and interaction effects (*P*>0.05) was indicated by ‘_’. Significant group effect is indicated with ‘G’. Significant speed effect is indicated with ‘S’. ‘$G \times S$’ indicated significant Group $\times$ Speed interaction. Means sharing similar letters differ significantly.

Table S3. Mean normalized absolute angular momentum for segments and whole body, and mean segmental and total cancellations.

|  | Preferred | | Fast | | Effect |
| --- | --- | --- | --- | --- | --- |
|  | Young | Old | Young | Old |  |
| ***Sagittal plane*** |  |  |  |  |  |
| Absolute H_trunk_*10^-3^ | 0.45 ± 0.12 | 0.60 ± 0.15 | 0.70 ± 0.16 | 0.96 ± 0.33 | G, S |
| Trunk cancellation (%) | 11.8 ± 4.10 | 10.6 ± 6.00 | 6.9 ± 2.90 | 8.6 ± 5.9 | S |
| Absolute H_legs_*10^-3^ | 3.35 ± 0.48^a^ | 3.70 ± 0.52^b^ | 4.98 ± 0.55^a^ | 4.81 ± 0.69^b^ | S, G $\times S$ |
| Legs cancellation (%) | 56.2 ± 4.60 | 54.0 ± 3.40 | 59.8 ± 2.70 | 55.7 ± 4.60 | G, S |
| Absolute H_arms_*10^-3^ | 0.14 ± 0.05 | 0.16 ± 0.04 | 0.28 ± 0.11 | 0.29 ± 0.12 | S |
| Arms cancellation (%) | 64.8 ± 7.80 | 60.8 ± 6.40 | 66.1 ± 9.40 | 60.7 ± 7.00 | G |
| Absolute H_total_*10^-3^ | 3.94 ± 0.61 | 4.47 ± 0.58 | 5.95 ± 0.75 | 6.07 ± 0.98 | S |
| Total cancellation (%) | 57.0 ± 4.80^a^ | 53.4 ± 4.20 | 61.5 ± 4.10^a,b^ | 53.9 ± 6.7^b^ | G, S, G $\times S$ |
| ***Frontal plane*** |  |  |  |  |  |
| Absolute H_trunk_*10^-3^ | 0.40 ± 0.15 | 0.48 ± 0.16 | 0.4 ± 0.14 | 0.5 ± 0.16 | - |
| Trunk cancellation (%) | 10.8 ± 7.00 | 8.80 ± 5.30 | 13.7 ± 5.60 | 12.7 ± 5.70 | S |
| Absolute H_legs_*10^-3^ | 1.03 ± 0.16 | 1.06 ± 0.13 | 0.98 ± 0.16 | 1.02 ± 0.12 | S |
| Legs cancellation (%) | 14.4 ± 5.9^a^ | 16.1 ± 7.3^b^ | 29.5 ± 7.5^a^ | 23.7 ± 90^b^ | S, G $\times S$ |
| Absolute H_arms_*10^-3^ | 0.11 ± 0.04 | 0.13 ± 0.03 | 0.18 ± 0.08 | 0.21 ± 0.10 | S |
| Arms cancellation (%) | 35.3 ± 12.7 | 34.5 ± 11.9 | 40.0 ± 9.7 | 40.8 ± 10.2 | S |
| Absolute H_total_*10^-3^ | 1.54 ± 0.31 | 1.67 ± 0.26 | 1.56 ± 0.30 | 1.72 ± 0.29 | - |
| Total cancellation (%) | 19.5 ± 8.80^a^ | 19.8 ± 8.80^b^ | 37.3 ± 7.20^a^ | 31.2 ± 9.90^b^ | S, G $\times S$ |
| ***Transversal plane*** |  |  |  |  |  |
| Absolute H_trunk_*10^-3^ | 0.23 ± 0.08 | 0.28 ± 0.05 | 0.38 ± 0.1 | 0.42 ± 0.12 | S |
| Trunk cancellation (%) | 7.10 ± 4.60 | 5.30 ± 3.00 | 6.80 ± 3.90 | 5.20 ± 3.00 | - |
| Absolute H_legs_*10^-3^ | 0.58 ± 0.1 | 0.62 ± 0.09 | 0.84 ± 0.16 | 0.82 ± 0.15 | S |
| Legs cancellation (%) | 11.3 ± 2.80 | 11.9 ± 2.80 | 15.9 ± 3.90 | 14.6 ± 3.4 | S |
| Absolute H_arms_*10^-3^ | 0.22 ± 0.08 | 0.25 ± 0.08 | 0.43 ± 0.20 | 0.45 ± 0.24 | S |
| Arms cancellation (%) | 29.6 ± 12.5 | 27.3 ± 6.80 | 15.1 ± 7.40 | 18.3 ± 7.30 | S |
| Absolute H_total_*10^-3^ | 1.03 ± 0.23 | 1.15 ± 0.18 | 1.65 ± 0.41 | 1.69 ± 0.42 | S |
| Total cancellation (%) | 44.2 ± 10.1 | 43.7 ± 8.1 | 56.0 ± 10.9 | 51.6 ± 10.3 | S |

Values are presented as mean ± SD.

Absolute angular momentum and cancellation were both average over the total duration of the stepping movement. Absolute segmental angular momentum was computed as the sum of absolute angular momenta of the bodies of the considered segment and normalized by body mass, body height and $\sqrt{g\cdot l}$ ($g$ = 9.81 m$\cdot$s^-2^ and $l$ = body height). Absolute angular momentum of the whole body (Absolute H_total_) was computed as the sum of absolute angular momenta of the 19 bodies and normalized by body mass, body height and $\sqrt{g\cdot l}$ ($g$ = 9.81 m$\cdot$s^-2^ and $l$ = body height). Segmental and total cancellation ($c$) was calculated according to Bennett et al.^5^:

$$c=\frac{\sum_{j=1}^{N} \left| H_{j} \right|-\left| \sum_{j=1}^{N} H_{j} \right|}{\sum_{j=1}^{N} \left| H_{j} \right|}$$

with $H_{j}$ is the angular momentum of the j-th body and N is the total number of the bodies of the considered segment.

Absence of group, speed and interaction effects (*P*>0.05) was indicated by ‘_’. Significant group effect is indicated with ‘G’. Significant speed effect is indicated with ‘S’. ‘$G \times S$’ indicated significant Group $\times$ Speed interaction. Means sharing similar letters differ significantly.

Table S4. Non normalized spatiotemporal parameters for the young and old participants in the preferred and fast speed conditions.

| **Parameters** | **Preferred** | | **Fast** | | **Effect** |
| --- | --- | --- | --- | --- | --- |
|  | Young | Old | Young | Old |  |
| Forward progression velocity ($m\cdot s^{-1})$ | 0.71 ± 0.16^a^ | 0.74 ± 0.11^b^ | 1.18 ± 0.19^a^ | 1.01 ± 0.21^b^ | S, G $\times S$ |
| Length of the 1^st^ Step ($m)$ | 0.59 ± 0.11 | 0.59 ± 0.08 | 0.74 ± 0.12 | 0.69 ± 0.14 | S |
| Width of the 1^st^ Step ($m)$ | 0.17 ± 0.02 | 0.17 ± 0.03 | 0.19 ± 0.03 | 0.17 ± 0.04 | S |
| Length of the 2^nd^ Step ($m)$ | 0.62 ± 0.12 | 0.63 ± 0.08 | 0.80 ± 0.12 | 0.74 ± 0.16 | S |
| Width of the 2^nd^ Step ($m)$ | 0.17 ± 0.03 | 0.15 ± 0.03 | 0.18 ± 0.04 | 0.15 ± 0.04 | G |
| 1^st^ Double support phase duration (s$)$ | 0.62 ± 0.09 | 0.52 ± 0.08 | 0.49 ± 0.08 | 0.44 ± 0.09 | G, S |
| 1^st^ Step execution phase duration (s$)$ | 0.43 ± 0.05 | 0.40 ± 0.05 | 0.34 ± 0.04 | 0.34 ± 0.05 | S |
| 2^nd^ Double support phase duration (s$)$ | 0.29 ± 0.07^a^ | 0.25 ± 0.05^b^ | 0.13 ± 0.03^a^ | 0.16 ± 0.03^b^ | S, G $\times S$ |
| 2^nd^ Step execution phase duration (s$)$ | 0.46 ± 0.05 | 0.49 ± 0.06 | 0.36 ± 0.04 | 0.38 ± 0.05 | G, S |
| Restabilisation phase duration (s$)$ | 1.42 ± 0.11 | 1.56 ± 0.16 | 1.42 ± 0.12 | 1.57 ± 0.18 | G |

Significant group effect is indicated with ‘G’. Significant speed effect is indicated with ‘S’. ‘$G \times S$’ indicated significant Group $\times$ Speed interaction. Means sharing similar letters differ significantly.
